# Race, ethnicity, and clinical outcome following sport-related concussion: a systematic review

**DOI:** 10.3389/fneur.2023.1110539

**Published:** 2023-06-14

**Authors:** Nathan E. Cook, Charles E. Gaudet, Alicia Kissinger-Knox, Brian C. Liu, Amy A. Hunter, Marc A. Norman, Altaf Saadi, Grant L. Iverson

**Affiliations:** ^1^Department of Physical Medicine and Rehabilitation, Harvard Medical School, Boston, MA, United States; ^2^Sports Concussion Program, MassGeneral Hospital for Children, Boston, MA, United States; ^3^Department of Physical Medicine and Rehabilitation, Spaulding Rehabilitation Hospital, Charlestown, MA, United States; ^4^Department of Public Health Sciences and Department of Pediatrics, University of Connecticut School of Medicine, Farmington, CT, United States; ^5^Injury Prevention Center, Connecticut Children's Medical Center and Hartford Hospital, Hartford, CT, United States; ^6^Department of Psychiatry, University of California, San Diego, San Diego, CA, United States; ^7^Department of Neurology, Harvard Medical School, Boston, MA, United States; ^8^Department of Neurology, Massachusetts General Hospital, Boston, MA, United States; ^9^Department of Physical Medicine and Rehabilitation, Schoen Adams Research Institute at Spaulding Rehabilitation, Charlestown, MA, United States

**Keywords:** concussion, clinical recovery, race, ethnicity, mild traumatic brain injury, social determinants of health (SDOH)

## Abstract

**Introduction:**

This systematic review examined whether race or ethnicity are associated with clinical outcomes (e.g., time to return to school/sports, symptom duration, vestibular deficits, and neurocognitive functioning) following sport-related concussion among child, adolescent, or college-aged student athletes. Additionally, this review assessed whether the existing literature on this topic incorporated or included broader coverage of social determinants of health.

**Methods:**

The online databases PubMed, MEDLINE^®^, PsycINFO^®^, CINAHL, Cochrane Library, EMBASE, SPORTDiscus, Scopus, and Web of Science were searched.

**Results:**

A total of 5,118 abstracts were screened and 12 studies met inclusion criteria, including 2,887 youth and young adults. Among the included articles, only 3 studies (25%) examined whether race and ethnicity were associated with outcomes following concussion as a primary objective. None of the studies assessed the association between social determinants of health and outcomes following concussion as a primary objective, although 5 studies (41.7%) addressed a social determinant of health or closely related topic as a secondary objective.

**Discussion:**

Overall, the literature to date is extremely limited and insufficient for drawing conclusions about whether race or ethnicity are categorically associated with outcomes from sport-related concussion, or more specifically, whether there are socioeconomic, structural, or cultural differences or disparities that might be associated with clinical outcome.

**Systematic review registration:**

identifier: PROSPERO, CRD42016041479, CRD42019128300.

## 1. Introduction

Health disparities associated with race and ethnicity are well established (1). A systematic review and technical report from the American Academy of Pediatrics characterized racial and ethnic child health disparities as “extensive, pervasive, and persistent” (2). There is evidence that disparities relating to both physical and mental health persist into college age (3–5). Disparities have been documented across the full spectrum of health and health care, including prevention, access to care, care utilization, and quality of care (2). Within the literature, race and ethnicity are sometimes used interchangeably, and these are not biological constructs. It is recognized that these terms serve as proxies of structural factors like racism, thereby encompassing a large range of social determinants of health. Social determinants of health are non-medical conditions in people's environments that affect their health, including the social constructs of race and ethnicity as well as other factors such as socioeconomic status, education, neighborhood environment, among others.

Racial and ethnic disparities are relevant to the evaluation, treatment, and rehabilitation of children, adolescents, and young adults following sport-related concussion. These disparities have been reported with regard to access and utilization of health care services for concussion (6–8), receipt of academic accommodations following concussion (9), and awareness of concussion signs and symptoms (10). Results from two emergency department (ED) surveillance datasets reporting on ED visits throughout the United States indicated that black youth were less likely to visit the emergency department for a head injury or concussion compared to non-Hispanic white youth (6, 8). Further, among youth presenting to the ED for a sport-related concussion, non-white youth were more likely to leave without being seen by a healthcare provider (11). Disparities have also been reported in terms of specialty care access, such that Hispanic youth were less likely to be seen for subspeciality concussion care, compared to subspecialty care for fractures (7). Following an ED visit for concussion, Hispanic children were significantly less likely to receive academic support compared to children who are not Hispanic (9). Taken together, these studies raise numerous concerns and questions about how social determinants may influence possible differences in access to health care services, service delivery, and in clinical outcomes.

The extent to which there are differences in clinical outcomes following sport-related concussion between individuals of various racial and ethnic identities is not well-understood. Clinical outcomes are diverse, such as the duration of symptoms, severity of neurocognitive deficits, time to return to school without accommodations, and time to return to sports. Identifying factors that are associated with worse, or better, clinical outcome following concussion, and quantifying the magnitude of those effects, is necessary to determine whether or the degree to which social determinants, health history, or injury characteristics should be included in prognostic risk models that could be used to guide medical management, treatment, and rehabilitation of injured student athletes as well as to identify potential foci for system-level change to reduce health disparities. A systematic review examining a broad array of potential factors that may be associated with clinical outcome following concussion was conducted in advance of the Concussion in Sport Group meeting in 2016 held in Berlin (12). This review identified 7 studies that analyzed whether clinical outcomes differed by race or ethnicity. Five of those studies reported null results and two studies reported an association (12). Given that the focus of this prior systematic review was extremely broad (i.e., investigating *any* potential predictor of concussion outcome and ultimately identifying 21 predictors), no individual potential risk factor was analyzed in depth, including race and ethnicity. Moreover, the literature search for this systematic review was conducted in June 2016 and many additional studies on concussion outcome have been published since that time. Therefore, an updated systematic review focused specifically on whether clinical outcomes (e.g., time to return to school/sports, symptom duration, vestibular deficits, neurocognitive functioning) following sport-related concussion differ in association with race or ethnicity is warranted. The objectives of this systematic review were to (i) determine if race or ethnicity are associated with clinical outcome following sport-related concussion among child, adolescent, or college-aged student athletes; (ii) examine whether the existing literature on this topic incorporates or includes broader coverage of social determinants of health; and (iii) identify knowledge gaps and directions for future research.

## 2. Materials and methods

### 2.1. Study eligibility and data sources

**Figure 1:**
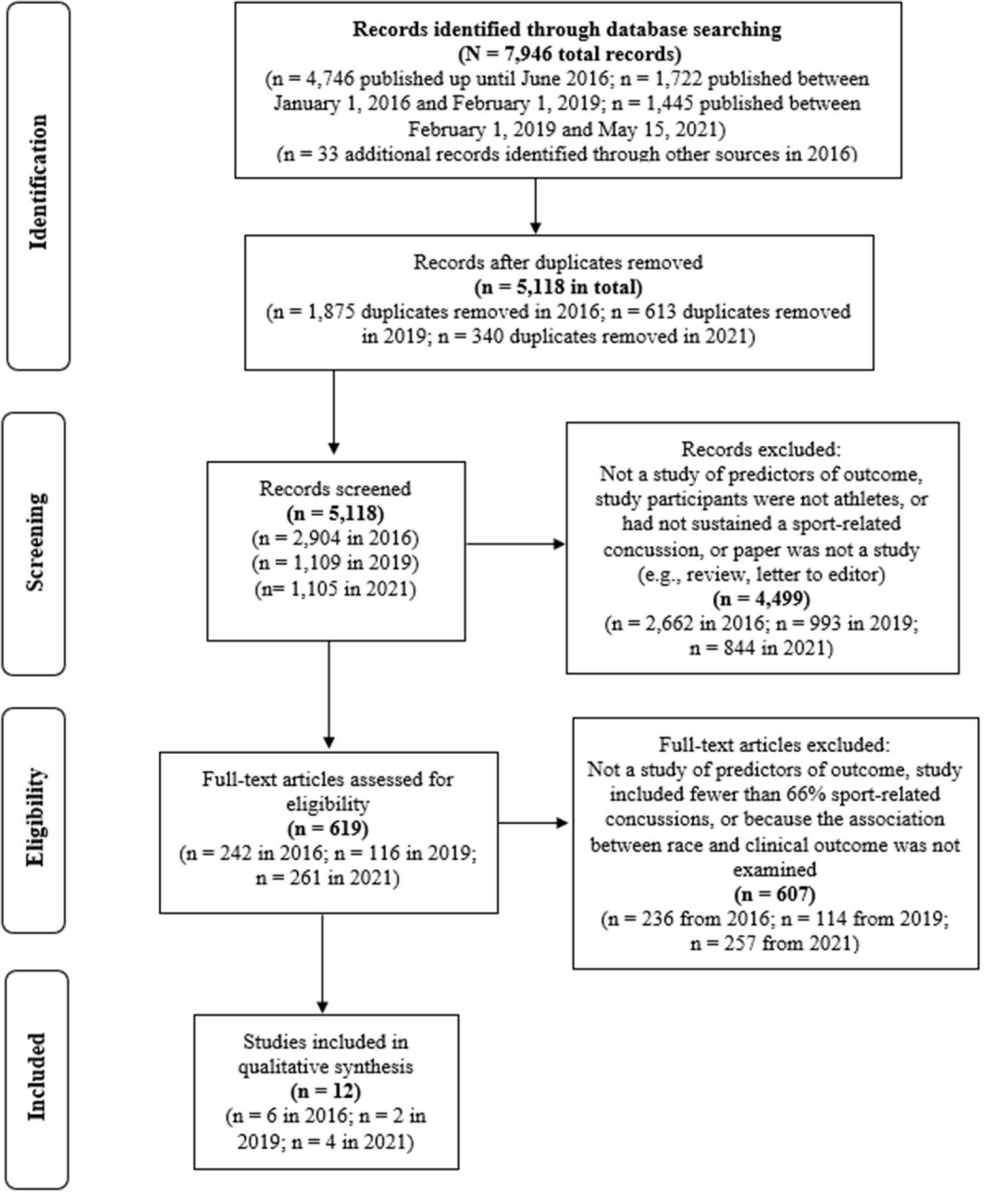
Systematic literature search (PRISMA). There was a mistake in the reporting of the number of records identified in the original search from 2016, as published by Iverson et al. (12). The initial search in 2016 yielded 4,746 records, as noted in this figure.

Articles were identified through online database searching, hand-searching reference lists (i.e., pearl growing), and performing cited reference searches (see Figure 1). Databases included PubMed, MEDLINE^®^, PsycINFO^®^, CINAHL, Cochrane Library, EMBASE, SPORTDiscus, Scopus, and Web of Science. Articles published in English from database inception to May 15, 2021 were included in our searches (Registration: PROSPERO CRD42016041479, CRD42019128300).

Two general categories relating to (i) sport and athlete-related terms, and (ii) brain concussion related terms were used as the key search terms, as follows: sport, sports [MeSH], athletic, athlete, player AND craniocerebral trauma, brain injuries, brain concussion, sports concussion, athletic injuries, mild traumatic brain injury, mTBI, traumatic brain injury, TBI, brain concussion, concussion, multiple concussions, repeated concussion, repetitive concussion, cumulative concussions, concussion history, brain damage, prognosis, outcome, recovery, risk factor, injury incidence, sex differences, gender, genetics, ApoE, BDNF, S100B, GFAP, severity, loss of consciousness, LOC, post-traumatic amnesia, PTA, amnesia, retrograde amnesia, seizure, seizures, learning disorder, ADHD, level of education, migraine, mental health, sleep disorders, medications, cervical injury, vestibular injury, psychological reactions, anxiety, depression, headaches, intractable headaches, magnetic resonance imaging, MRI, computer tomography, and CT. This extraordinarily broad set of search terms was used to replicate the terms used in our prior systematic reviews (12–16) because many articles examine multiple predictors, and race/ethnicity might simply be included as a secondary or demographic variable, and thus, a more focused search would likely miss articles that did in fact examine race/ethnicity as one of many predictors or variables analyzed.

### 2.2. Definition of clinical outcomes

To be included, clinical outcomes needed to be measured one or more weeks following injury. We did not study acute clinical outcome. Outcome and clinical recovery were defined broadly to include self-reported resolution of concussion symptoms, improvement in neuropsychological functioning, improvement in vestibular deficits, and time to medical clearance and return to play and/or participation in normal activities, such as school.

### 2.3. Data extraction

Three authors (AKK, CG, and BL) completed the extraction of information from the articles [i.e., first author, year of publication, the PubMed identification number, number of participants, proportion of female participants, age, setting, time since injury, characteristics of the index concussion (e.g., proportion experiencing loss of consciousness, posttraumatic amnesia), study design, outcome examined, and the period for assessing the clinical outcome]. Authors extracted details regarding the association between race and outcome from the index concussion, including results of statistical significance testing, direction of effects, and any quantitative metrics reported in the study (e.g., recovery times, parameter estimates from statistical modeling, effect sizes). Discrepancies were resolved by discussion.

For the included studies, three authors (AKK, CG, and BL) coded whether social determinants of health (SDoH) and associated subcategories were reflected. A coding sheet was developed by the study team to identify SDoH and associated subcategories derived from both the Healthy People 2020 (https://www.healthypeople.gov/2020/topics-objectives/topic/social-determinants-of-health) and the Healthy People 2030 websites (https://health.gov/healthypeople/objectives-and-data/social-determinants-health). The five SDoH domains identified by Healthy People were included: *Economic Stability, Education Assess and Quality, Health Care Access and Quality, Neighborhood and Built Environment*, and *Social and Community Context*. Healthy People 2020 provided a list of several key issues and underlying factors associated with each of the five domains. For example, an important issue included under Economic Stability is “poverty.” All the key issues and underlying factors were included as separate variables to code, and the list was cross-referenced with material on the Healthy People 2030 website, where a separate webpage is available for each SDoH domain. Three co-authors (NC, AKK, and BL) read the text descriptions and identified other key issues or underlying factors that were not included in the Healthy People 2020 bulleted list.

The same three authors (AKK, CG, and BL) also collected information about the sample size, average age, age range, gender, racial composition, and ethnic composition of the sample. The extent to which the studies analyzed or provided information about key health equity variables (e.g., culture/acculturation, socioeconomic status, and language) was characterized as follows: the study (i) provided no mention of the variable, (ii) included the variable as a demographic category only, or (iii) examined the variable in depth (e.g., the health equity variable was a primary variable of interest in the study, or outcome or prognostic results were stratified and reported across levels of the variable). We also coded race and ethnicity in this manner. The content analysis coding sheet is included in the Supplementary material.

### 2.4. Risk of bias and level of evidence

Risk of bias for each article was rated by three authors (NC, AKK, and CG) using the Newcastle-Ottawa Scale, a risk of bias assessment system designed for observational studies (17). Differences in risk of bias ratings were resolved by discussion. The scale includes nine items assessing specific study features relating to Sample Selection, Comparability between Research Groups, and Outcome (for Cohort studies) or Exposure (for Case Control studies). For each of the nine items, credit is assigned for stronger, higher quality design features based on criteria presented on the scoring sheets and in the coding manual (17). This scale has been reported to be suitable for systematic reviews (18). For the current review, when rating the Newcastle-Ottawa Scale, we considered race or ethnicity as the “exposure” and prolonged recovery or worse clinical outcome (e.g., symptom duration ≥28 days, worse neurocognitive test performance, longer duration of return to play) from the index concussion as the “outcome.” Additionally, the level of evidence for each article was rated by the same three authors (NC, AKK, and CG) using the Oxford Classification for Evidence-Based Medicine. Differences in the level of evidence ratings were resolved by discussion.

### 2.5. Narrative synthesis

Substantial heterogeneity in the participant populations, comparators, clinical outcomes, and recovery time periods precluded quantitative synthesis and analyses. We performed a narrative synthesis and descriptively summarized the study samples and methodologies. We summarized the results of statistical significance testing (using each study authors' definition of significance, most often was *p* < 0.05) regarding whether clinical outcome from concussion was or was not associated with race or ethnicity, including the direction of effects. We reported the results of our content analysis descriptively.

## 3. Results

### 3.1. Search results and study characteristics

A total of 5,118 abstracts were screened, 619 full-text articles were evaluated for inclusion, and 12 studies met inclusion criteria (19–30). Four studies (25%) reported concussion outcome among children or adolescents (maximum sample age ≤ 19 years) (19–22). Eight studies (75%) reported outcome among college-age samples (i.e., the mean sample age >18, they included participants age ≥ 20 years, or they indicated the sample consisted exclusively of college athletes) (23–30). Characteristics for the study populations are summarized in Table 1. There were 2,887 participants (mean age = 17.8 years, SD = 2.04, median = 18.9 years among the eight studies that reported sample age). Two studies involved overlapping samples (19, 20)—the number of non-overlapping participants was 2,769. Ten (83.3%) of the studies were published since 2015, and 5 (41.7%) were published since 2018. One study was published in 2010 and one study was published in 2004. Studies included a median of 26.0% girls (range 0%-50.8%). The racial and ethnic compositions of the included studies are summarized in Table 2. Two-thirds of the studies (66.7%) were prospective designs and one-third (33.3%) were retrospective. Studies in this review examined the following outcomes: recovery time/symptom duration (k = 6) (21, 22, 24–26, 30), time to return to sports (k = 5) (19, 20, 26, 27, 30), mental health problems (k = 2) (28, 29), and neuropsychological functioning and symptom severity (k = 1) (23) following concussion [several studies reported multiple outcomes, so the number of studies sums to greater than 12].

**Table 1:** Summary of the study outcomes (studies listed alphabetically).

**Author (Year)**	***N***	**% female**	**Age (M, SD, range)**	**Design, setting, outcome**	**Characteristics of most recent (index) concussion**						**Duration of follow-up**
					**Time since injury**	**% LOC**	**% AOC**	**% PTA**	**% RTA**	**Mechanism**	
Aggarwal et al. (19)	118	28.8%	15.8 (1.5), 13–19	Design: Retrospective Cohort Setting: Specialty clinic Outcome: Time to return to play or normal activities	Within 10 days; 0–5 days = 79% 5–10 days = 29%	NR	NR	NR	NR	89% sport; also MVA, falls, non-sport collision	Until recovery; Md time to recovery= 17 days
Aggarwal et al. (20)	227	25.5%	15.73 (1.49), 13–19	Design: Retrospective Cohort Setting: Specialty clinic Outcome: Time to return to play or normal activities	Within 10 days; 0-5 days = 72%; 5–10 days = 28%	NR	NR	NR	NR	90.3% sport; also MVA, falls, non-sport collision	Until recovery; M = 67.2 days, Md = 33 days
Asplund et al. (30)	101	9.90%	18.8 (NR), 14–23	Design: Prospective Cohort Setting: Specialty clinic Outcome: Symptom duration, presence of clinical signs, and time to return to play	NR	10%	NR	33%	17%	100% Sport	NR (category of >7 days or < 7 days)
Kontos et al. (23)	96	18.75%	19.3 (2.08) 14–23	Design: Prospective Case-Control Setting: Sports Outcome: Neurocognitive performance and symptom reporting	2 days and 7 days post injury	1%	NR	2%	NR	100% Sport	7 days
Madura et al. (24)	40	27.5%	19.96 (6.28), NR	Design: Prospective Cohort Setting: Sports Outcome: Symptom duration	NR	NR	NR	NR	NR	36 Sport; 4 from “recreation”	Until full return to play clearance; < 20 days or >20 days
McDevitt et al. (25)	87	26.44%	19.47 (6.02), NR	Design: Prospective Case-Series Setting: Specialty clinic Outcome: Symptom duration	NR	22.99%	NR	NR	NR	100% Sport	Until full return to play clearance; < 60 days or >60 days M = 55.73; SD = 85.79
Mihalik et al. (26)	45	0%	NR	Design: Prospective Cohort Setting: Collegiate sports Outcome: Symptom duration and time to return to play	< 6 h post injury	NR	NR	NR	NR	100% Sport; Football	Until unrestricted RTP; Md SRT = 6.1 days (IQR 5.8, *n* = 45) Md RTP = 12.3 days (IQR 7.8, *n* = 36)
Morgan et al. (21)	120	50.8%	14.8 (2.0), 9-18	Design: Retrospective Case-Control Setting: Specialty clinic Outcome: Symptom duration	NR	21.7%	NR	30.8%		100% Sport	Until recovery; PCS: symptoms >3 months Control: symptom resolution 3 weeks
Pattinson et al. (27)	127	23.62%	18.9 (1.3), 17-23	Design: Prospective Cohort Setting: Collegiate sports Outcome: Time to return to play	Within 21 h post injury	5.5%	NR	18.9%	NR	100% Sport	Until recovery; Md RTS = 12.9 (8.7–20.7) days
Thomas et al. (22)	1.733	32.5%	NR (NR), 10–17	Design: Retrospective Cohort Setting: Specialty clinic Outcome: Symptom duration	Within 30 days of injury	13.4%	NR	NR	NR	100% Sport and Recreational activities	Until symptom resolution; Md = 17 days
Vargas et al. (28)	126	31.75%	Concussed: 18.4 (0.8), NR Control: 18.9 (0.9), NR	Design: Prospective Case-Control Setting: Specialty clinic Outcome: Symptoms of depression	6.6 (8.7), 1–41 days	NR	NR	NR	NR	100% Sport	Up to 41 days
Yang et al. (29)	67	25.35%	NR	Design: Prospective Cohort Setting: Collegiate sports Outcome: Symptoms of depression and anxiety	1 week post injury	NR	NR	NR	NR	100% Sport	Up to 12 months

AOC, Alteration of Consciousness; LOC, Loss of Consciousness; PTA, Post-Traumatic Amnesia; RTA, Retrograde Amnesia; NR, Not reported; M, mean; SD, standard deviation; Md, median; IQR, Interquartile range; HR, Hazard ratio; OR, Odds ratio, CI, Confidence interval.

**Table 2:** Summary of review findings regarding race, ethnicity, and outcome from concussion (studies listed alphabetically).

**First author (year)**	**Racial and ethnic composition of the sample**	**Differential outcome primary focus of the study?**	**Addressed or discussed health inequity?**	**Addressed or discussed any social determinants of health?**	**Statistically significant association between race/ethnicity and outcome?**
Aggarwal et al. (19)	White: 72 (61%), Hispanic: 28 (24%), Black Non-Hispanic: 18 (15%)	Yes	No	Yes	No
Aggarwal et al. (20)	White: 122 (53.7%), Hispanic: 67 (29.5%), African American: 38 (16.5%)	Yes	No	Yes	Yes; Hispanic and African American adolescents recovered faster—also, Hispanic and African American youth were more likely to be in the lower SES quartile and have public insurance
Asplund et al. (30)	Black: 23 (23.2%), White: 73 (73.7%), Hispanic: 3 (3.0%)	No	No	No	No
Kontos et al. (23)	White: 48 (50%), African American: 48 (50%)	Yes	No	Yes	Yes; African American student athletes were significantly more likely to have at least one clinically significant decline on ImPACT at 7 days post concussion and scored lower at 7 days post compared to their baseline on processing speed
Madura et al. (24)	White: 20 (50.0%), African American: 8 (20.0%), Latino: 2 (5.0%), Native American: 1 (2.5%), Asian/Pacific Is.: 1 (2.5%), Multiracial: 2 (5.0%), Not reported: 6 (15.0%)	No	No	No	No
McDevitt et al. (25)	Caucasian: 45 (52%), African American: 12 (14%), Hispanic/Latino: 3 (3%), Native American: 1 (1%), Asian/Pacific Islander: 2 (2%), Multiracial: 2 (2%), Not Reported: 22 (25%)	No	No	No	No
Mihalik et al. (26)	Non-Hispanic White: 18 (40.0%), Black: 16 (35.6%), Other: 11 (24.4%)	No	No	No	Mixed findings; African American student athletes did not differ from non-Hispanic White youth (either symptom resolution time or return to play time), the small subgroup of student athletes who identified as other race (*n* = 11) took longer to experience symptom resolution compared to non-Hispanic White student athletes, although they did not differ on time to return to play
Morgan et al. (21)	Black: 22 (18.3%), Caucasian: 96 (80.0%), Unknown: 2 (1.7%)	No	Yes	Yes	No
Pattinson et al. (27)	White: 82 (64.6%), African American: 24 (18.9%), Asian: 8 (6.3%), Hawaiian or Pacific Islander: 2 (1.6%), Multiple: 10 (7.9%), Unknown/Not Reported: 1 (0.8%)	No	No	No	No
Thomas et al. (22)	White: 1,459 (79.3%), Black/African American: 223 (12.1%), Other: 158 (8.6%)	No	No	Yes	Mixed findings; No significant differences in recovery by 2, 3, or 4 weeks—White youth were more likely to take >4 weeks to recover
Vargas et al. (28)	White: 86 (68.3%), African American: 27 (21.4%), Asian American: 3 (2.4%), Latin American: 1 (0.8%), Multiracial: 8 (6.3%), Other: 1 (0.8%)	No	No	No	Yes; Student athletes who identified as either African American, multiracial, or other race experienced more depressive symptoms following concussion compared to White student athletes (medium effect size), although overall levels of depression symptoms were mild and generally fell below the threshold for clinical significance
Yang et al. (29)	White: 52 (73.2%), Non-White: 19 (26.8%) (in relation to the number of concussions)	No	No	No	No

### 3.2. Level of evidence and risk for bias assessment

Level of Evidence and Newcastle-Ottawa Scale ratings are summarized in Table 3. The mean level of evidence was 3.3 (SD = 0.5). Nine studies (75%) were rated as level 3 (i.e., cohort studies) (19, 20, 22, 24–27, 29, 30) and 3 studies (25%) as level 4 (i.e., case-control studies or lower-quality prognostic cohort studies) (21, 23, 28). Studies received credit for between three to eight Newcastle-Ottawa Scale items (mean = 5.7, SD = 1.3).

**Table 3:** The Newcastle-Ottawa Quality Assessment Scale scores and the level of evidence of the included studies (listed alphabetically).

**First author (year)**	Design^*^	**Newcastle-Ottawa scale** ^**∧**^				**Center of evidence-based medicine**
		**Selection (0–4)**	**Comparability (0–2)**	**Outcome/ exposure (0–3)**	**Total credits**	**Level of evidence (1–5)**
Aggarwal et al. (19)	Cohort	✩★✩★	★★	✩★★	6	3
Aggarwal et al. (20)	Cohort	✩★★★	★★	★★★	8	3
Asplund et al. (30)	Cohort	★★✩★	✩✩	★★✩	5	3
Kontos et al. (23)	Case-Control	★✩★★	★✩	★★✩	7	4
Madura et al. (24)	Cohort	✩★✩★	★✩	★★✩	5	3
McDevitt et al. (25)	Cohort	✩★✩★	★✩	★★✩	5	3
Mihalik et al. (26)	Cohort	✩★★★	★✩	★★✩	6	3
Morgan et al. (21)	Case-Control	★✩★✩	✩✩	★★★	5	4
Pattinson et al. (27)	Cohort	✩★★★	✩✩	★★★	6	3
Thomas et al. (22)	Cohort	✩★★★	★★	★★✩	7	3
Vargas et al. (28)	Case-Control	★✩★✩	✩✩	✩★✩	3	4
Yang et al. (29)	Cohort	✩★★★	✩✩	★★✩	5	3

* For the NOS, we determined the study design in reference to the determination of whether race or ethnicity is a predictor of worse clinical outcome—not necessarily based on the original study design. ^∧^When completing the NOS, we rated the study in relation to considering race or ethnicity as a predictor of worse clinical recovery from concussion. Thus, in certain circumstances the original study design (which might have been examining many predictors of recovery), might have earned credit on one of the NOS parameters, but when we examined the study in relation to its ability to determine if race or ethnicity are associated with worse outcome from a concussion, the study might not have earned credit on that parameter. ✩ Credit was not allotted for that item. ★ Credit was allotted for that item.

### 3.3. Race, ethnicity, and clinical outcomes following concussion

Across all studies and outcomes, the most frequent finding was that clinical outcomes did not differ based on race or ethnicity. Most studies (58.3%) did not find a statistically significant association between race or ethnicity and outcome from concussion. Three studies (25.0%) reported a statistically significant result (20, 23, 28) and the remaining two studies (16.7%) reported predominantly null results with an isolated statistically significant finding (22, 26). Of note, among these studies with at least one statistically significant result, two (40%) reported that racial and ethnic minority youth had better outcomes (i.e., faster recovery times, less likely to take >4 weeks to recover) (20, 22). The remaining three studies reported that racial and ethnic minority youth had worse outcomes (i.e., worse subacute neurocognitive test performance, higher levels of depressive symptoms, and longer symptom resolution time) (23, 26, 28). Among the included articles, only three studies (25%) were specifically designed and carried out to examine whether race and ethnicity were associated with clinical outcomes following concussion (19, 20, 23), and two of these studies reported statistically significant results (20, 23) while the other study reported a null result. However, there are major caveats and considerable nuance to appreciate and consider when interpreting results from these studies. Further detail about the individual studies and their findings are provided in sections below.

### 3.4. Studies of children and adolescents

Four studies were identified that examined the potential association between race and outcome from a sport-related concussion in children and adolescents (19–22), and two of those studies reported on a partially overlapping sample (19, 20). Thus, there were three independent samples totaling 2,080 youth studied. All three pediatric samples were recruited from specialty concussion clinics.

The clinical outcome in all four studies was recovery time. This included days to recovery for two studies (19, 20), recovery within several time periods for one study (i.e., within 2 weeks, 3 weeks, 4 weeks, or >4 weeks) (22), and persistent symptoms lasting >3 months following an injury in the fourth study (21). Two studies with partially overlapping samples reported mixed results. An initial study of 118 youth noted a 4 day difference in median recovery time between youth identified as White race (*n* = 72, median 17 days) compared to youth identified as “Minority” race (*n* = 46, median 13 days), and this difference was not statistically significant in either univariate (*p* = 0.06) or multivariate (*p* = 0.50) analyses (19). The investigators added 109 additional youth to the sample and replicated the analyses. With the larger sample (*N* = 227), the authors noted an eight day difference in median recovery time between youth identified as White race (*n* = 122, median 19 days) compared to youth identified as a combined Hispanic/African American group (*n* = 105, median 11 days), and this difference was statistically significant in univariate analyses (*p* < 0.001) (20). In multivariate models, the authors further stratified their racial and ethnic analyses by gender. Compared to girls who identified as White race (the reference group in these analyses), neither girls who identified as Hispanic or African American (combined; *p* = 0.91) nor boys who identified as White (*p* = 0.08) differed in recovery time, though boys who identified as Hispanic or African American (combined) recovered significantly *sooner* following injury (*p* = 0.003) (20).

A large study of 1,733 patients (79.3% White) found that youth identified as White were more likely to have prolonged recovery (symptom resolution >4 weeks post injury), compared to youth identified as Black/African American (12.1% of the sample) or Other race (8.6% of the sample; further detail about the racial identities of these youth was not reported) (22). Of note, this study also reported null findings; the racial groups did not differ in terms of proportions of youth recovered within 2 weeks, 3 weeks, or 4 weeks (22). A retrospective case-control study from a specialty concussion clinic compared 40 youth diagnosed with “post-concussion syndrome” (i.e., symptoms lasting >3 months following injury) to matched controls (matched on age and sex) who recovered within 3 weeks (21). The prolonged recovery group included 90% White and 10% Black youth, and the controls included 75.0% White and 22.5% Black youth as well as 2.5% with “unknown” race; these differences were not statistically significant (*p* = 0.13) (21). Of note, the prolonged recovery group contained only four youth identified as Black.

### 3.5. Studies of college students

Eight studies were identified that examined the potential association between race and ethnicity and outcome from sport-related concussion among college-aged individuals (23–30). Across the eight studies there were a total of 689 young adults studied. Study settings included primary care sports medicine clinics, an outpatient hospital-based concussion program, and multiple National Collegiate Athletics Association (NCAA) institutions.

#### 3.5.1. Time to return to play

Of the five studies that examined return to play time for college-aged individuals (24–27, 30), none revealed a statistically significant difference in time to return to sport times associated with race or ethnicity. Specifically, in a study of 101 collegiate athletes, researchers reported no statistically significant difference in time to return to play following concussion between student athletes identifying as Black, White, or Hispanic (30). Notably, return to play was assessed as a binary outcome, with prolonged recovery defined as more than seven days to return to play (30). In a case series of 87 athletes (age: M = 19.47, SD = 6.02) return to play time (prolonged return to play defined as more than 60 days) did not differ between racial groups (25). Similarly, in a study of 40 student athletes (age: M = 19.96, SD = 6.28), there were no differences in prolonged return to play (taking >20 days to obtain clearance for full return) associated with race (24). A study of 127 collegiate athletes did not show an association between race and time to return to sports (prolonged recovery was defined as requiring 14 days or more to return to sport) (27). Furthermore, return to play times, assessed as a continuous variable, did not differ by race among 45 collegiate athletes following concussion (26).

#### 3.5.2. Symptom severity and duration

One study of 45 collegiate athletes examined symptom severity and duration. Univariate analyses revealed those whose race or ethnicity was classified as “other” experienced longer symptom resolution time as compared to Non-Hispanic White athletes. However, there were no differences in symptom duration between Non-Hispanic White and Black athletes, as well as no association between race and symptom severity (26).

#### 3.5.3. Neurocognitive functioning

There were mixed findings regarding neurocognitive functioning. Results of a case-control study that matched 48 African American and 48 White high school and collegiate athletes (age, years: M = 19.33, SD = 2.08) revealed that African American athletes were more likely to have clinically significant declines on computerized neurocognitive testing, relative to preinjury baseline performance, at seven days post-injury compared to White participants (*p* = 0.03). Additionally, relative to preinjury baseline, African American athletes showed greater declines in processing speed than White athletes at seven days post injury (*p* = 0.01) (23). In contrast, a study of 45 collegiate athletes that compared neurocognitive functioning between Non-Hispanic White, Black, and athletes grouped as “other,” did not reveal an association between race and neurocognitive performance, as measured using the Standardized Assessment of Concussion at multiple time points following injury (26).

#### 3.5.4. Vestibular functioning

In a prospective cohort study of 45 collegiate athletes (51 total concussions), athletes whose race or ethnicity was categorized as “other” performed worse on balance testing compared to White athletes, as measured by the Balance Error Scoring System in an unadjusted negative binomial regression model. However, there were no differences in balance testing performance between Non-Hispanic White and Black athletes (26).

#### 3.5.5. Psychological functioning

In a study of 84 collegiate athletes, athletes who did not identify as White reported greater levels of depressive symptoms following injury (*p* = 0.01) but did not differ in baseline depressive symptoms (*p* = 0.16) or change in depressive symptoms (*p* = 0.29), as measured by the 7-item Beck Depression Inventory-Fast Screen. Notably, when history of psychiatric treatment was controlled for, race was not associated with depressive symptoms following injury (*p* = 0.38) (28). Similarly, race was not associated with symptoms of depression or anxiety at 1 week following injury in a study of 67 collegiate athletes, as measured by the Center for Epidemiological Studies Depression Scale and State Trait Anxiety Inventory. Athletes with a pre-existing history of depression were more likely to experience depressive symptoms and state anxiety following concussion (29).

### 3.6. Social determinants of health and health equity

Among the studies included in this review, none were designed and carried out considering social determinants of health (SDoH) as a guiding framework. Further, no study specifically referenced or discussed social determinants of health. With that said, results of our content analysis revealed that five studies (41.7%) addressed a SDoH or closely related topic in some form or fashion (19–23). One study addressed three SDoH domains (21), one study addressed two domains (19), and the remaining three studies addressed one domain (20, 22, 23). The most commonly addressed domain was Healthcare Access and Quality (k = 3) (19, 21, 22). Two studies addressed Economic Stability (19, 20). Two other studies addressed Education Access and Quality (21, 23). One study addressed Social and Community (21). No studies addressed the Neighborhood and Built Environment domain.

No studies directly examined or studied health equity. Only one study referenced or discussed the topic of health equity. Specifically, in their discussion section, Morgan and colleagues position their findings in the context of considerable literature demonstrating childhood health disparities associated with race (21).

### 3.7. Demographic or health factors as exclusionary criteria

Four studies (33.3%) excluded participants based on demographic or health factors (19, 20, 23, 28). They were not excluded based on race. Participants were most commonly excluded for learning disabilities (23), psychiatric disorders (19, 20, 23), or a history of inpatient treatment (28), or substance use disorders (23, 28).

### 3.8. Social determinants or health equity as future clinical and research considerations

Five studies (41.7%) discussed future directions and research needs that implicated or were related in some form or fashion to social determinants or health equity (19, 20, 23, 26, 28). In terms of SDoH domains, social and community (19, 20, 23, 26, 28), economic stability (19, 23), and education access and quality (19, 23) were most commonly implicated in the context of future clinical and research directions.

## 4. Discussion

Race and ethnicity are social constructs, not biological attributes (31–33). We identified 12 published studies that examined whether there was an association between race or ethnicity and worse clinical outcome from sport-related concussion. We sought to determine whether there is a general association between race or ethnicity and outcome from this injury, and the extent to which other sociodemographic factors or social determinants of health might be associated with any such identified differences. Importantly, of those 12 studies, only three were designed specifically to examine race, ethnicity, or both as a primary variable of interest. In the studies reviewed, the association between race and outcome, broadly, has not been well-characterized, when race was included, it was generally considered a demographic variable of possible interest, and it sometimes was combined (or conflated) with ethnicity. Moreover, the sample sizes associated with self-identified race or ethnicity were often too small to have reasonably powered analyses. Overall, the literature to date is extremely limited and insufficient for drawing conclusions about whether race or ethnicity are categorically associated with outcome from this injury, or more specifically, whether or which social determinants of health might be associated with clinical outcome.

Some of the studies in this review used samples from specialty clinics. In a recent specialty concussion care study, there were no racial disparities in how injured youth athletes were initially medically managed or how they ultimately presented to the specialty clinic despite racial differences in school type and insurance coverage (34). Specialty clinic visits for concussion care during the pandemic were substantially less, however, than prior to the pandemic, and patients tended to present considerably later following injury (i.e., a delayed presentation for specialty care) (35). There is evidence that youth who present for specialty care for concussion earlier have better clinical outcomes, as measured by faster clinical recovery or being less likely to have prolonged symptoms (36–38). Therefore, given disparities in access to specialty care, those disparities could partially underlie differential clinical outcomes.

Even when individuals may have access to healthcare, perceptions and racial inequalities within the healthcare setting may make minoritized populations less likely to seek out healthcare providers. In many cases, the providers are not demographically similar to the patients they serve, and this can create, for example, barriers of understanding and willingness to work collaboratively. Consequently, this may adversely impact healthcare outcomes. Future researchers using specialty clinic samples are encouraged, if possible, to contextualize who is seen in the clinic relative to the local population. For example, if the proportion of the local population is 45% Black but the clinic sample is only 5% Black, reporting this discrepancy will help contextualize possible insurance-related or other access-related issues and barriers to specialty care. Of course, researchers are encouraged to directly study social determinants of health like socioeconomic factors, health literacy, and community-level factors that might be associated with recruitment bias, access to care, and outcome from injury.

There are some studies illustrating differences associated with race on some clinical measures used to assess the effects of, and monitor recovery from, concussion (20, 39). There are many possible reasons underlying some of these differences, including quality of education (40), socioeconomic status (39–41), and stereotype threat (42). Stereotype threat refers to “being at risk of confirming, as self-characteristic, a negative stereotype about one's group” [(42), p. 797]. Stereotype threat, contextual variables, and perceived discrimination are associated with lower scores on neuropsychological testing in some studies (43–46). Moreover, it is possible that stereotype threat can be a social barrier in a health care clinical encounter that can contribute to greater patient anxiety and possible disparities in health care utilization (47). More research designed to deconstruct and quantify the associations between a broad range of contextual and interpersonal factors that might influence reporting or performance on clinical outcome measures, or how they are interpreted, will likely lead to more refined and personalized use of these measures.

Some research has identified differences and disparities associated with race or ethnicity with regard to awareness of concussion signs and symptoms (10), utilization of health care services for concussion (6–8), and receiving academic accommodations following concussion (9). Awareness of concussion signs and symptoms facilitates immediate removal from play following concussion and is associated with better clinical outcome in college students (48). College students who continue to participate in sports following concussion, in contrast to immediate removal from play, are more likely to have greater symptoms, longer recovery times, and greater time away from sports (49, 50). Therefore, when athletes, athletic trainers, and coaches have less knowledge and awareness about concussion signs and symptoms, injured athletes might be less likely to be removed from play following an injury—thus increasing their risk for prolonged recovery. Health care service utilization is also associated with outcome.

## 5. Future directions

Future concussion researchers are encouraged to discuss differences associated with race contextualized within a social ecological model, and it is important to appreciate that racism and other social determinants of health, not race *per se*, are risk factors for different health outcomes. Racism is a social determinant of health that operates at the intrapersonal, interpersonal, institutional, and structural levels contributing to health inequities and worse health outcomes for children and adolescents (51). Race is therefore sometimes used as a proxy for vulnerability to institutional and structural racism, socioeconomic disparities, and health inequities. There is excellent guidance for researchers on reporting race and ethnicity in medical and science journals that is designed to promote fairness, equity, consistency, and clarity (31).

Researchers and healthcare providers are encouraged to address systemic inequities in research and training. Myths and miseducation appear persistent in medical training. For example, 25% of surveyed residents believed that black skin is thicker than white, and there were misbeliefs that Black people experience pain differently than whites (52). This has led to racial biases in pain management, as well as more systemic inequalities in medical treatment (53–55). Such racial biases have been identified among counseling and clinical psychology trainees as well. For example, given identical symptomatology, graduate students rated a hypothetical Black client as less symptomatic compared to a hypothetical White client (56). Moreover, those from minoritized populations may not feel understood by providers, and their concerns may be dismissed by misperceptions, racial biases, and language barriers.

There are also important considerations regarding family structures. For example, some parents may have limited ability to take time off from work and thus to attend numerous healthcare appointments, perhaps especially those located some distance or time from their home or work. Other youth may be in the care of grandparents, and thus attention to caregiver demographics and family-specific factors potentially impacting healthcare may be the key.

Telehealth is a strategy for improving access and quality of pediatric healthcare (57). Telehealth is possible for youth sport-related concussions (58, 59), and in pediatric neurology more broadly (60). In a pilot study comparing in-person to telehealth concussion care, therapeutic alliance scores for parents and caregivers were significantly higher for the in-person condition than for the telehealth condition—but not for the patients themselves. Both telehealth and in-person care were associated with high satisfaction scores (61). However, in a large pediatric neurology study, there were racial and ethnic disparities between youth who used telephone encounters vs. telemedicine (audio and video) encounters (60). In some cases, families do not have the technology (e.g., smart phones) or the technological literacy to participate in telemedicine. More research is also needed in regard to disparities and outcomes associated with telehealth concussion care.

## 6. Conclusion

Based on the present review, it is not known the extent to which there are differences in clinical outcome from sport-related concussion associated with race or ethnicity—or the possible underlying reasons for any such differences. Identifying and quantifying factors that are associated with worse, or better, clinical outcome following concussion will help inform future multivariable prognostic risk models that could be used to guide medical management and inform personalized and precision treatment and rehabilitation for injured student athletes. Moreover, identifying these factors might help guide efforts to improve health literacy, enhance equitable access to health care, and reduce disparities in health outcomes.

## Data availability statement

The original contributions presented in the study are included in the article/Supplementary material, further inquiries can be directed to the corresponding author.

## Author contributions

NC helped conceptualize the review, drafted sections of the manuscript, extracted data from all studies in the review, rated risk of bias and level of evidence for all studies in the review, assisted with the literature review, edited the manuscript, and approved the final manuscript. CG, AK-K, and BL extracted data from studies in the review, rated risk of bias and level of evidence for studies in the review, edited the manuscript, and approved the final manuscript. AH and AS reviewed the articles included in the review for conceptual and methodological issues and edited the manuscript. MN critically reviewed and edited the manuscript. GI helped conceptualize the review, conducted article screening prior to the full text reviews, drafted sections of the manuscript, and edited drafts of the manuscript. All authors approved the final manuscript as submitted and agree to be accountable for all aspects of the work.
