# Supplementary material for: Race, ethnicity, and clinical outcome following sport-related concussion: a systematic review
Source: Front Neurol. 2023 Jun 14;14:1110539. doi: 10.3389/fneur.2023.1110539 (PMC10306165; doi:10.3389/fneur.2023.1110539)
Supplement: Supplementary file 1 [file Data_Sheet_1.pdf]

Race, Ethnicity, and Clinical Outcome Following Sport-Related Concussion: A Systematic Review

Supplementary Table 1. Quotes relating to social determinants of health

First Author	SDoH Domain	SDoH Subcategory	Page Number	Article Section	Column/Location	Quote
Aggarwal (2019) (1)	Economic Stability	Ability to afford healthcare	7	Discussion	Right	"Our analysis indicated that SES factors such as insurance type may influence concussion recovery. Minorities in this sample were more likely than Whites to be from lower income households and have public insurance (Medicaid, CHIP) or student athletic insurance. Our finding that insurance was a predictor of shorter resolution times is consistent with a recent study that determined that middle school, high school, and collegiate athletes who had private insurance took longer to return to school than those with public health insurance (Zuckerman et al., 2017)."
Aggarwal (2019) (1)	Health Care Access	Health and/or dental insurance	3	Data Collection Procedure	Right	"Health insurance that paid for the participant's health provider concussion visit was classified as follows: none, public (e.g., Medicaid, Children's Health Insurance Program [CHIP]), student athletic insurance, and private (commercial)."
Aggarwal (2020) (2)	Economic Stability	Ability to afford health care	812	Introduction	Right	"…we determined that public health insurance (e.g., Medicaid/Children's Health Insurance Program [CHIP]) was associated with shorter concussion recovery times."
Kontos (2010) (3)	Education Access	Whether children are from low-income families	736	Introduction	Middle	"Among the factors that have been proposed by researchers to influence neurocognitive performance differences among racial/ethnic groups are education and reading level... SES, or more specifically the quality of education, has been reported by researchers (e.g., Manly et al., 2002) to play a significant confounding role in reported differences in neurocognitive performance among racial/ethnic groups. Preliminary interpretations of recent studies suggest that lower SES is negatively related to neurocognitive achievements (Noble, Norman, & Farah, 2005)."
Kontos (2010) (3)	Education Access	Literacy	741	Discussion	Middle	"The cognitive reserve hypothesis may offer some explanation of why education and literacy levels seem to decrease the gap in neurocognitive performance between different racial and cultural groups (Stern, Albert, Tang, & Tsai, 1999)."

First Author	SDoH Domain	SDoH Subcategory	Page Number	Article Section	Column/Location	Quote
Kontos (2010) (3)	Social and Community Context	Societal attitudes and norms	742	Discussion	Middle	"Another confounding factor that may have limited the assessment of neurocognitive performance in the current study is the level of acculturation. African Americans who are less acculturated (to other, in this case, White cultural values, beliefs, and practices) may be less familiar with potential content and context in neurocognitive tests and therefore perform lower and at slower speeds on such tests than their more acculturated counterparts"–pg. 742, discussion
Morgan (2015) (4)	Education Access	Whether children experience social discrimination	593	Results	Left	"Significant stressors (close family member deaths and bullying) during SRC recovery was reported by 3 (7.5%) PCS patients."
Morgan (2015) (4)	Health Care Access	Health and/or dental insurance	591	Results	Right	"Neither race (p=0.134) nor type of insurance (p >0.999) was associated with PCS."
Morgan (2015) (4)	Social and Community Context	Depression or anxiety in family caregivers	590	Intro	Left	"Predictors of PCS after mTBI in children are increasing parental anxiety, parental financial resources, preexisting learning difficulties, psychiatric illness, family stressors, symptom attribution, and a child's decreasing health-related quality of life."
Morgan (2015) (4)	Social and Community Context	Positive versus negative relationships at home	590	Intro	Left	"Predictors of PCS after mTBI in children are increasing parental anxiety, parental financial resources, preexisting learning difficulties, psychiatric illness, family stressors, symptom attribution, and a child's decreasing health-related quality of life."
Morgan (2015) (4)	Health Care Access	Access to primary care	593	Discussion	Right	"We also did not find an association between race and development of PCS. This finding was notable because children of minority race are significantly more likely to experience underdiagnosis; undertreatment; and conditions like asthma, attention deficit hyperactivity disorder, and learning difficulties. In addition, a prospective, observational study of 71 adults with mTBI who sought care at an emergency department found that although patients were discharged with instructions to follow up with a primary care provider within 1–2 weeks, African Americans were less likely to do so (OR = 0.36, 95% CI 0.13–0.99)."
Thomas (2018) (5)	Health Care Access and Quality	Access to healthcare	62	Discussion	Left	"Delayed symptom presentation or seeking medical care from a nonspecialist first may lead to longer recovery observed in this study… Our work, in line with others, suggests that to minimize risk of prolonged recovery, pediatric patients should receive specialist care as soon as possible postconcussions so that providers can implement appropriate cognitive and physical rest measures to hasten recovery."

Study Coding Results Relating to Health Equity Variables

Aggarwal (2019)(1)

**Health Equity Variables**	*No mention*	*Demographic category only*	*Examined in depth*
Race	□	□	X
Ethnicity	□	□	X
Culture	X	□	□
Socioeconomic Status	□	□	X
Language	□	X	□

**Exclusionary Criteria**	*Yes*	*No*
Are participants excluded based on demographic or sociocultural or health factors?	X	□
**Future Directions**	*Yes*	*No*
Any future directions/research needs regarding social determinants or health equity discussed?	X	□

Aggarwal (2020)(2)

**Health Equity Variables**	*No mention*	*Demographic category only*	*Examined in depth*
Race	□	□	X
Ethnicity	□	□	X
Culture	X	□	□
Socioeconomic Status	□	□	X
Language	□	X	□

**Exclusionary Criteria**	*Yes*	*No*
Are participants excluded based on demographic or sociocultural or health factors?	X	□
**Future Directions**	*Yes*	*No*
Any future directions/research needs regarding social determinants or health equity discussed?	X	□

Asplund (2004)(6)

**Health Equity Variables**	*No mention*	*Demographic category only*	*Examined in depth*
Race	□	□	X
Ethnicity	□	□	X
Culture	X	□	□
Socioeconomic Status	X	□	□
Language	X	□	□

**Exclusionary Criteria**	*Yes*	*No*
Are participants excluded based on demographic or sociocultural or health factors?	□	X
**Future Directions**	*Yes*	*No*
Any future directions/research needs regarding social determinants or health equity discussed?	□	X

Kontos (2010)(3)

**Health Equity Variables**	*No mention*	*Demographic category only*	*Examined in depth*
Race	□	□	X
Ethnicity	X	□	□
Culture	X	□	□
Socioeconomic Status	X	□	□
Language	X	□	□

**Exclusionary Criteria**	*Yes*	*No*
Are participants excluded based on demographic or sociocultural or health factors?	X	□
**Future Directions**	*Yes*	*No*
Any future directions/research needs regarding social determinants or health equity discussed?	X	□

Madura (2016)(7)

**Health Equity Variables**	*No mention*	*Demographic category only*	*Examined in depth*
Race	□	□	X
Ethnicity	□	□	X
Culture	X	□	□
Socioeconomic Status	X	□	□
Language	X	□	□

**Exclusionary Criteria**	*Yes*	*No*
Are participants excluded based on demographic or sociocultural or health factors?	□	X
**Future Directions**	*Yes*	*No*
Any future directions/research needs regarding social determinants or health equity discussed?	□	X

McDevitt (2015)(8)

**Health Equity Variables**	*No mention*	*Demographic category only*	*Examined in depth*
Race	□	□	X
Ethnicity	□	□	X
Culture	X	□	□
Socioeconomic Status	X	□	□
Language	X	□	□

**Exclusionary Criteria**	*Yes*	*No*
Are participants excluded based on demographic or sociocultural or health factors?	□	X
**Future Directions**	*Yes*	*No*
Any future directions/research needs regarding social determinants or health equity discussed?	□	X

Mihalik (2020)(9)

**Health Equity Variables**	*No mention*	*Demographic category only*	*Examined in depth*
Race	□	□	X
Ethnicity	X	□	□
Culture	X	□	□
Socioeconomic Status	X	□	□
Language	X	□	□

**Exclusionary Criteria**	*Yes*	*No*
Are participants excluded based on demographic or sociocultural or health factors?	□	X
**Future Directions**	*Yes*	*No*
Any future directions/research needs regarding social determinants or health equity discussed?	X	□

Morgan (2015)(4)

**Health Equity Variables**	*No mention*	*Demographic category only*	*Examined in depth*
Race	□	□	X
Ethnicity	X	□	□
Culture	X	□	□
Socioeconomic Status	□	□	X
Language	X	□	□

**Exclusionary Criteria**	*Yes*	*No*
Are participants excluded based on demographic or sociocultural or health factors?	□	X
**Future Directions**	*Yes*	*No*
Any future directions/research needs regarding social determinants or health equity discussed?	□	X

Pattinson (2020)(10)

**Health Equity Variables**	*No mention*	*Demographic category only*	*Examined in depth*
Race	□	□	X
Ethnicity	□	□	X
Culture	X	□	□
Socioeconomic Status	X	□	□
Language	X	□	□

**Exclusionary Criteria**	*Yes*	*No*
Are participants excluded based on demographic or sociocultural or health factors?	□	X
**Future Directions**	*Yes*	*No*
Any future directions/research needs regarding social determinants or health equity discussed?	□	X

Thomas (2018)(5)

**Health Equity Variables**	*No mention*	*Demographic category only*	*Examined in depth*
Race	□	□	X
Ethnicity	X	□	□
Culture	X	□	□
Socioeconomic Status	X	□	□
Language	X	□	□

**Exclusionary Criteria**	*Yes*	*No*
Are participants excluded based on demographic or sociocultural or health factors?	□	X
**Future Directions**	*Yes*	*No*
Any future directions/research needs regarding social determinants or health equity discussed?	□	X

Vargas (2015)(11)

**Health Equity Variables**	*No mention*	*Demographic category only*	*Examined in depth*
Race	□	□	X
Ethnicity	□	X	□
Culture	X	□	□
Socioeconomic Status	X	□	□
Language	X	□	□

**Exclusionary Criteria**	*Yes*	*No*
Are participants excluded based on demographic or sociocultural or health factors?	X	□
**Future Directions**	*Yes*	*No*
Any future directions/research needs regarding social determinants or health equity discussed?	X	□

Yang (2015)(12)

**Health Equity Variables**	*No mention*	*Demographic category only*	*Examined in depth*
Race	□	□	X
Ethnicity	X	□	□
Culture	X	□	□
Socioeconomic Status	X	□	□
Language	X	□	□

**Exclusionary Criteria**	*Yes*	*No*
Are participants excluded based on demographic or sociocultural or health factors?	□	X
**Future Directions**	*Yes*	*No*
Any future directions/research needs regarding social determinants or health equity discussed?	□	X

References

1. Aggarwal SS, Ott SD, Padhye NS, Meininger JC, Armstrong TS. Clinical and demographic predictors of concussion resolution in adolescents: A retrospective study. Appl Neuropsychol Child. 2019;8(1):50-60.
2. Aggarwal SS, Ott SD, Padhye NS, Schulz PE. Sex, race, ADHD, and prior concussions as predictors of concussion recovery in adolescents. Brain Inj. 2020;34(6):809-17.
3. Kontos AP, Elbin RJ, Covassin T, Larson E. Exploring differences in computerized neurocognitive concussion testing between African American and White athletes. Archives of Clinical Neuropsychology. 2010;25:734-44.
4. Morgan CD, Zuckerman SL, Lee YM, King L, Beaird S, Sills AK, et al. Predictors of postconcussion syndrome after sports-related concussion in young athletes: a matched case-control study. Journal of neurosurgery Pediatrics. 2015;15(6):589.
5. Thomas DJ, Coxe K, Li H, Pommering TL, Young JA, Smith GA, et al. Length of Recovery From Sports-Related Concussions in Pediatric Patients Treated at Concussion Clinics. Clin J Sport Med. 2018;28(1):56-63.
6. Asplund CA, McKeag DB, Olsen CH. Sport-related concussion: factors associated with prolonged return to play. Clin J Sport Med. 2004;14(6):339-43.
7. Madura SA, McDevitt JK, Tierney RT, Mansell JL, Hayes DJ, Gaughan JP, et al. Genetic variation in SLC17A7 promoter associated with response to sport-related concussions. Brain Inj. 2016;30(7):908-13.
8. McDevitt J, Tierney RT, Phillips J, Gaughan JP, Torg JS, Krynetskiy E. Association between GRIN2A promoter polymorphism and recovery from concussion. Brain Inj. 2015;29(13-14):1674-81.
9. Mihalik JP, Chandran A, Powell JR, Roby PR, Guskiewicz KM, Stemper BD, et al. Do Head Injury Biomechanics Predict Concussion Clinical Recovery in College American Football Players? Annals of Biomedical Engineering2020. p. 2555-65.
10. Pattinson CL, Meier TB, Guedes VA, Lai C, Devoto C, Haight T, et al. Plasma Biomarker Concentrations Associated with Return to Sport following Sport-Related Concussion in Collegiate Athletes-A Concussion Assessment, Research, and Education (CARE) Consortium Study. JAMA Network Open. 2020;3:1-11.
11. Vargas G, Rabinowitz A, Meyer J, Arnett PA. Predictors and prevalence of postconcussion depression symptoms in collegiate athletes. J Athl Train. 2015;50(3):250-5.
12. Yang J, Peek-Asa C, Covassin T, Torner JC. Post-concussion symptoms of depression and anxiety in division I collegiate athletes. Dev Neuropsychol. 2015;40(1):18-23.
